# Diagnostic accuracy of Node-RADS for lymph node metastasis in rectal cancer: a systematic review and meta-analysis

**DOI:** 10.3389/fonc.2026.1899172

**Published:** 2026-08-26

**Authors:** Tian Tian, Yang Li, Linfang Xu, Zhihua Lu

**Affiliations:** Department of Radiology, The Fourth Affiliated Hospital of Soochow University, Suzhou Dushu Lake Hospital, Medical Center of Soochow University, Suzhou, Jiangsu, China

**Keywords:** diagnostic accuracy, lymph node metastasis, magnetic resonance imaging, meta-analysis, Node-RADS, rectal cancer

## Abstract

**Objective:**

To systematically evaluate the diagnostic accuracy and clinical value of the Node Reporting and Data System (Node-RADS) for lymph node metastasis in rectal cancer (RC).

**Methods:**

The study followed PRISMA-DTA guidelines and was registered in PROSPERO (CRD420261372147). PubMed, Embase, Web of Science, Cochrane Library, CNKI, Wanfang, VIP, and the Chinese Biomedical Literature Database were searched from inception to April 2026. Two reviewers independently screened studies, extracted data, and assessed methodological quality using QUADAS-2. Sensitivity, specificity, positive likelihood ratio (PLR), negative likelihood ratio (NLR), diagnostic odds ratio (DOR), and the summary receiver operating characteristic (SROC) area under the curve (AUC) were pooled using a bivariate random-effects model. Heterogeneity, publication bias, and clinical utility were assessed using subgroup and sensitivity analyses (including exclusion of the post-neoadjuvant chemoradiotherapy cohort), Deeks’ funnel plot, and Fagan’s nomogram based on the observed prevalence.

**Results:**

Seven studies involving 994 patients with RC were included, comprising 374 with and 620 without lymph node metastasis. Pooled sensitivity, specificity, PLR, NLR, and DOR were 0.82 (95% confidence interval [CI], 0.74-0.87), 0.84 (95% CI, 0.73-0.91), 5.15 (95% CI, 3.06-8.68), 0.22 (95% CI, 0.16-0.30), and 23.66 (95% CI, 12.92-43.34), respectively; the SROC AUC was 0.88 (95% CI, 0.85-0.91). Excluding the clinically distinct post-neoadjuvant chemoradiotherapy study yielded sensitivity of 0.79 (95% CI, 0.73-0.84), specificity of 0.90 (95% CI, 0.84-0.94), and AUC of 0.88 (95% CI, 0.85-0.90), while specificity heterogeneity decreased from 89.07% to 69.79%. At the observed prevalence of 37.6%, a positive Node-RADS result increased the post-test probability to approximately 76%, whereas a negative result reduced it to approximately 12%. No significant funnel-plot asymmetry was detected, although publication bias could not be excluded because only seven studies were included.

**Conclusion:**

Node-RADS shows good diagnostic accuracy and clinical discriminatory ability for lymph node metastasis in RC and may serve as a standardized imaging-based adjunct for preoperative nodal assessment. However, substantial heterogeneity, *post hoc* threshold selection, and limited external validation indicate that standalone use requires further prospective validation.

**Systematic review registration:**

https://www.crd.york.ac.uk/PROSPERO/View/CRD420261372147, identifier CRD420261372147.

## 1 Introduction

Rectal cancer (RC) stands as a prevalent malignant tumor of the digestive system. In recent years, colorectal cancer has continued to have high incidence and mortality rates worldwide, with a persistently increasing disease burden. Early diagnosis, accurate staging, and individualized treatment have always been the focus of clinical attention (1). Lymph node metastasis represents one of the most common metastatic patterns of RC and is an important factor affecting clinical staging, treatment selection, and prognosis. For individuals at risk of lymph node metastasis, precise preoperative appraisement of lymph node status helps determine the need for neoadjuvant therapy, optimize surgical approaches, and guide decisions regarding postoperative adjuvant therapy. Inaccurate assessment of lymph node status may lead to undertreatment or overtreatment, thereby affecting individuals’ long-term survival and quality of life (2).

Currently, magnetic resonance imaging (MRI) represents a vital imaging modality for preoperative local staging and lymph node assessment in RC. The diagnosis of lymph node metastasis by conventional MRI mainly relies on features such as lymph node size, morphology, margin, and signal intensity. However, relying solely on short diameter or conventional morphological criteria has certain limitations. Small lymph nodes may still harbor metastases, whereas enlarged nodes are not invariably malignant (3, 4). Recent node-matched MRI-pathology evidence also indicates that current size and morphological criteria may be insufficiently accurate for nodal staging (5).

To harmonize the imaging appraisement of lymph nodes in tumor patients, researchers have developed the Node Reporting and Data System 1.0 (Node-RADS 1.0). It is currently a standardized imaging reporting system for assessing the risk of tumor-associated lymph node involvement on computed tomography (CT) and MRI images. Node-RADS integrates imaging features like lymph node size, internal architecture, margin, and shape, aiming to reduce subjective variability among observers and improve the standardization, reproducibility, and clinical interpretability of imaging reports (6). Subsequently, Node-RADS has been gradually applied in the appraisement of lymph nodes in various tumors and different anatomical sites. A recent narrative review has summarized an overall trend in which higher Node-RADS scores are associated with a greater probability of nodal metastasis, with several studies reporting better diagnostic performance than short diameter alone (7).

In recent years, the application of Node-RADS in the preoperative appraisement of lymph node metastasis of RC has gained increasing attention. Niu et al. have evaluated the diagnostic value of Node-RADS for mesorectal lymph node metastasis of RC based on MRI data, and results have uncovered that Node-RADS achieves an area under the curve (AUC) of 0.862 for predicting pathological node-positive (pN+) status, outperforming the European Society of Gastrointestinal and Abdominal Radiology (ESGAR) and short-diameter criteria. This finding suggests good diagnostic performance for preoperative lymph node evaluation in RC (8). Furthermore, Jiang et al. have compared the diagnostic efficacy of Node-RADS across distinct imaging modalities, including contrast-enhanced CT, T2WI, and contrast-enhanced T1WI. Their results have demonstrated that Node-RADS is superior to short-diameter measurement in all modalities, with no significant differences in diagnostic efficacy among the modalities, indicating that the system has certain cross-modality applicability (9).

Although the above studies suggest that Node-RADS has certain diagnostic value for lymph node metastasis in RC, existing studies still vary in terms of study design, sample size, imaging modality, positivity cutoff values of Node-RADS, patients receiving neoadjuvant therapy or not, and diagnostic performance results. Findings from a single study are insufficient to fully reflect its overall diagnostic accuracy. A recent systematic review and meta-analysis evaluated the diagnostic performance of Node-RADS across different tumor types and anatomical sites (10). However, that review included different tumor types and anatomical sites, and evidence specifically focusing on the RC population remains relatively limited. Thus, the current study intended to carry out a systematic review and meta-analysis to comprehensively retrieve relevant studies on Node-RADS for diagnosing lymph node metastasis of RC, thereby systematically appraising the diagnostic accuracy and clinical application value of Node-RADS in the preoperative diagnosis of lymph node metastasis of RC, and offering evidence for clinical decision-making.

## 2 Methods

### 2.1 Protocol and registration

The present study was implemented as per the Preferred Reporting Items for Systematic Reviews and Meta-Analyses of Diagnostic Test Accuracy Studies (PRISMA-DTA) guidelines (11). The protocol was registered with the International Prospective Register of Systematic Reviews (PROSPERO) (registration number CRD420261372147).

### 2.2 Literature search

PubMed, Embase, Web of Science, Cochrane Library, CNKI, Wanfang, VIP, and the Chinese Biomedical Literature Database were retrieved from their inception until April 2026. English search terms included “rectal cancer”, “rectal neoplasm”, “rectal tumor”, “Node-RADS”, and “Node Reporting and Data System”. Medical subject headings (MeSH) and free-text terms were combined for search. The basic retrieval strategy was (rectal cancer OR rectal carcinoma OR rectal neoplasm OR rectal tumor) AND (Node-RADS OR Node Reporting and Data System), and it was adjusted according to the search rules of each database. The detailed search strategies are provided in **Supplementary Material 1**. Moreover, the reference lists of the included studies were manually examined to avoid any omission.

### 2.3 Inclusion and exclusion criteria

The inclusion criteria were outlined below: (1) the study participants were individuals with pathologically or clinically confirmed RC; (2) the index test was Node-RADS based on CT or MRI; (3) the reference standard was postoperative pathological diagnosis or pathological assessment; (4) the study outcomes included at least sensitivity, specificity, and AUC, or true positives (TP), false positives (FP), false negatives (FN), and true negatives (TN) could be acquired or calculated from the original data; (5) the study design was a diagnostic accuracy study, including cross-sectional, retrospective, or cohort studies; (6) the study subjects were human. The exclusion criteria were: (1) reviews, meta-analyses, conference abstracts, case reports, expert consensus, guidelines, or commentary articles; (2) studies whose participants were not individuals with RC, or studies with data on RC not extractable separately; (3) studies for which the full texts were inaccessible or data were severely absent and could not be obtained after contacting the authors; (4) duplicate publications or studies with overlapping study populations, in which case the study with the most complete data or the largest sample size was retained.

### 2.4 Literature screening and data acquisition

Two investigators (Tian Tian and Li Yang) independently screened the literature using EndNote according to the inclusion and exclusion criteria. They first reviewed titles and abstracts and then performed full-text assessment. The following information was extracted: first author, publication year, country, study design, sample size, age, sex, imaging modality, treatment setting, reference standard, TP, FP, FN, TN, sensitivity, and specificity. To investigate methodological heterogeneity and reproducibility, we additionally extracted MRI field strength and slice thickness, CT reconstruction thickness and contrast phase, nodal compartment, analysis level (patient-level or node-level), reader number and experience, blinding and adjudication procedures, and inter-observer agreement when reported. If diagnostic 2 × 2 data were not directly provided, they were independently derived by the two reviewers. Disagreements were resolved through discussion with a third investigator (Xu Linfang).

### 2.5 Quality assessment of the included studies

The quality of the included studies was appraised utilizing the quality assessment of diagnostic accuracy studies 2 (QUADAS-2) tool in RevMan 5.4. This tool appraised the risk of bias and applicability of each included study. Any divergences between the two investigators were addressed through discussion with a third investigator (Xu Linfang).

### 2.6 Statistical methods

Statistical analyses were performed using Stata 15.0. TP, FP, FN, and TN were obtained from the included studies or calculated from the original data, and diagnostic 2 × 2 tables were constructed. A bivariate random-effects model was used to jointly pool sensitivity and specificity and to estimate PLR, NLR, DOR, and their 95% CIs. A summary receiver operating characteristic (SROC) curve was generated to derive the AUC. A threshold effect was assessed using Spearman correlation between logit sensitivity and logit (1-specificity), with P<0.05 indicating a potential threshold effect. Heterogeneity was evaluated using Cochran’s Q and I² statistics. I²>50% or P<0.05 indicated substantial heterogeneity. Subgroup analyses were performed by imaging modality (CT and MRI), but were interpreted descriptively because the number of studies per subgroup was small and multimodal estimates from Jiang et al. arose from the same patient cohort. Univariate meta-regression included tumor-location reporting, pathological T-stage reporting, direct radical surgery, and use of a validation set. Imaging-acquisition and reader-related variables were summarized descriptively rather than entered into meta-regression because they were incompletely reported, showed little between-study variation, or were available in too few studies for reliable modeling. Model robustness was assessed through goodness-of-fit, bivariate normality, influence, and outlier analyses. In addition, a separate sensitivity analysis excluded Yang et al. (2026), because that study enrolled patients after neoadjuvant chemoradiotherapy and evaluated a clinically distinct post-treatment setting. Publication bias was assessed using Deeks’ funnel plot, recognizing its limited power with fewer than 10 studies. Fagan’s nomogram was constructed using the observed prevalence of lymph node metastasis in the included population (374/994, 37.6%; rounded to 38% for plotting) rather than an external assumed prevalence. All tests were two-sided, and P<0.05 was considered statistically significant.

## 3 Results

### 3.1 Literature search and inclusion

**Figure 1:**
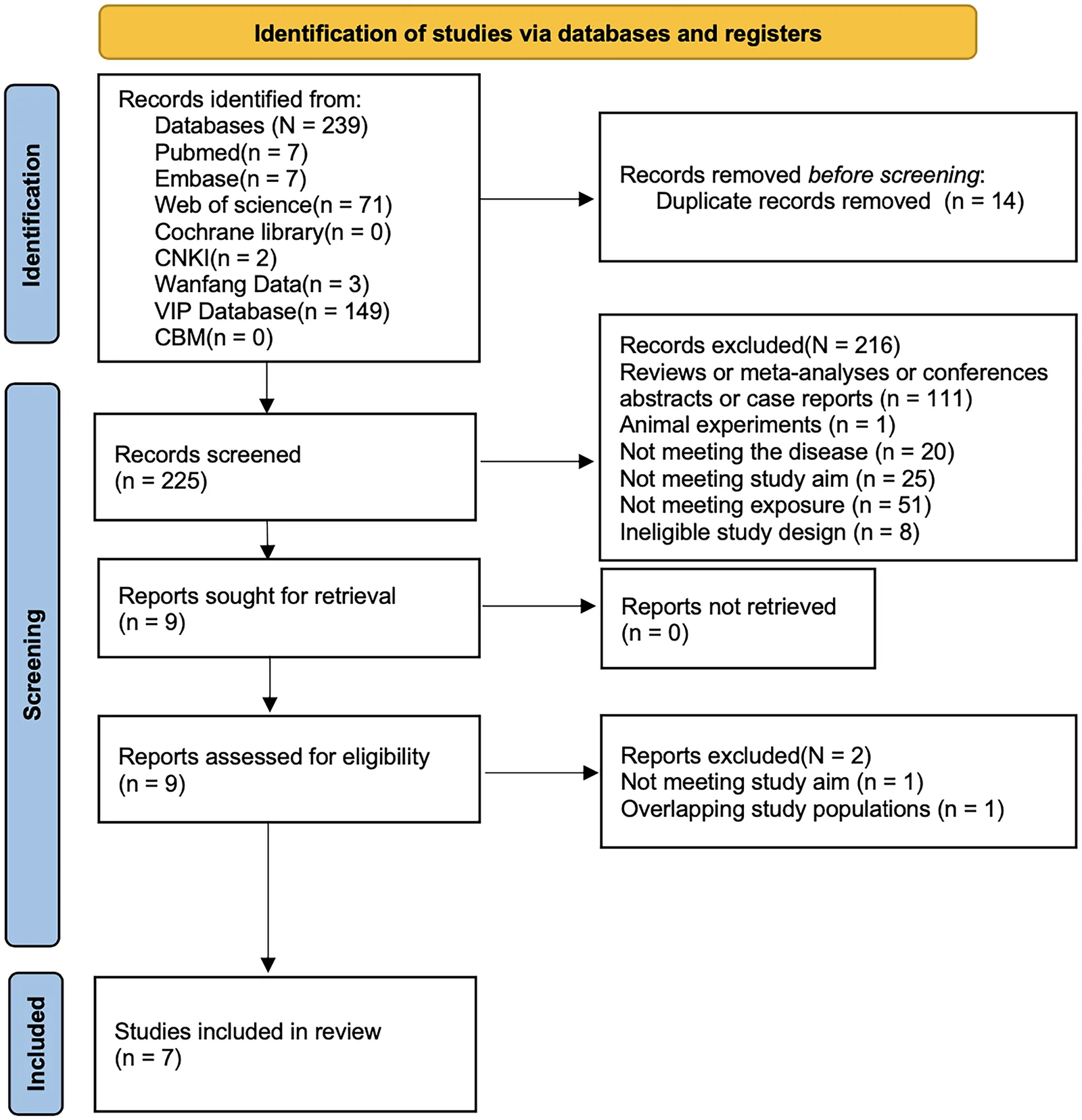
PRISMA 2020 flow diagram of study selection.

Totally 239 records were identified. After the removal of duplicates using EndNote, 225 records remained. Following preliminary screening by reviewing titles and abstracts, studies that were unrelated to the topic, reviews, conference abstracts, case reports, or non-RC articles were removed. Afterwards, full-text studies were reviewed for further screening, and studies that did not use Node-RADS, did not allow separate extraction of RC data, did not permit construction of fourfold tables, or had duplicate data were eliminated. Finally, 7 studies were included (8, 9, 12–16), comprising 6 English articles and 1 Chinese article. The screening process is detailed in **Figure 1**.

### 3.2 Data extraction and quality appraisement of the included studies

**Figure 2:**
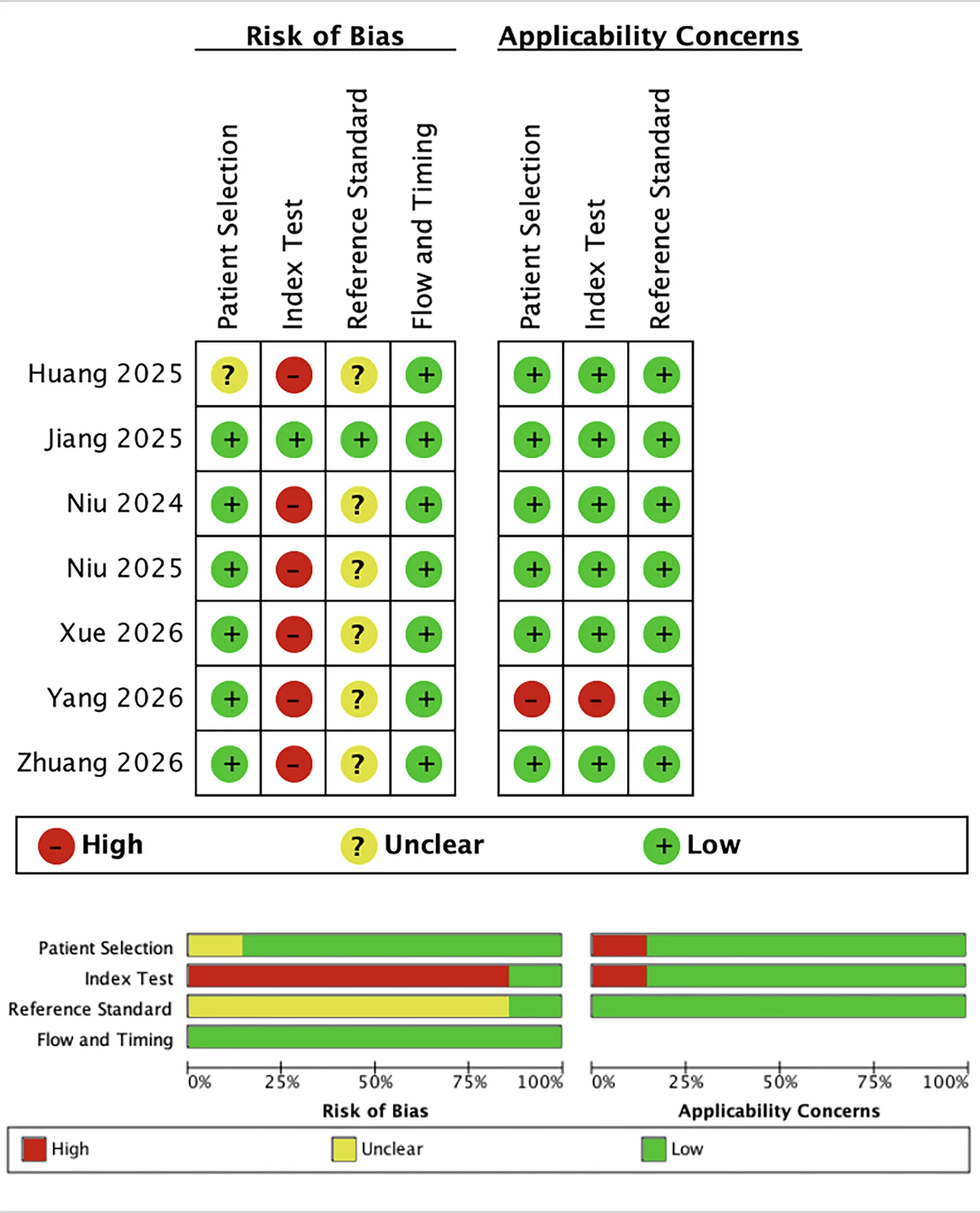
Risk of bias and applicability concerns of the included studies assessed using the QUADAS-2 tool.

All seven included studies were retrospective and enrolled 994 patients with RC. Among them, 62.88% were male, the weighted mean age was 62.27 years, and 374 (37.6%) had lymph node metastasis while 620 did not. The basic characteristics are shown in **Table 1**. As shown in **Figure 2**, the QUADAS-2 assessment indicated generally low concern for flow, timing, and applicability, but six index-test judgments were rated as high risk of bias, primarily because Node-RADS positivity thresholds were not prespecified and were instead selected *post hoc* using ROC curves or the Youden index. Yang et al. (2026) also had high applicability concerns for patient selection and the index test because it enrolled patients with locally advanced RC after neoadjuvant chemoradiotherapy and evaluated a deep learning radiomics Node-RADS framework. These features differed from conventional pretreatment Node-RADS assessment.

**Table 1:** Characteristics of the included studies.

Study	Year	Country	Study design	Study period	Sample size	Age, years	Male/female	Treatment setting	Imaging modality	Reference standard
Jiang et al. (9)	2025	China	Retrospective study	Jun 2017–Dec 2023	113	66.0 ± 10.5	73/40	No neoadjuvant therapy; direct radical surgery	CT, MRI	Postoperative pathology
Niu et al. (12)	2024	China	Retrospective study	Jul 2022–Aug 2023	146	60.2 ± 10.6	90/56	No neoadjuvant therapy; direct radical surgery	CT	Postoperative pathology
Niu et al. (8)	2025	China	Retrospective study	Jul 2022–Sep 2023	154	60.4 ± 10.6	93/61	No neoadjuvant therapy; direct radical surgery	MRI	Postoperative pathology
Xue et al. (13)	2026	China	Retrospective study	May 2019–Apr 2023	163	62.6 ± 10.1	98/65	No neoadjuvant therapy; direct radical surgery	MRI	Postoperative pathology
Yang et al. (15)	2026	China	Retrospective multicenter study	Jan 2018–Mar 2025; Mar 2021–Nov 2024; Nov 2019–Dec 2023	190	59.8 ± 10.1	127/63	After neoadjuvant chemoradiotherapy; TME surgery	MRI	Postoperative pathology
Zhuang et al. (14)	2026	China	Retrospective study	Jun 2017–Dec 2023	126	67.0 ± 8.9	82/44	No neoadjuvant therapy; direct radical surgery	MRI	Postoperative pathology
Huang et al. (16)	2025	China	Retrospective study	Dec 2022–Dec 2023	102	62.2 ± 9.7	62/40	No neoadjuvant therapy; direct radical surgery	MRI	Postoperative pathology

Values are presented as mean ± standard deviation unless otherwise indicated. CT, computed tomography; MRI, magnetic resonance imaging; nCRT, neoadjuvant chemoradiotherapy; TME, total mesorectal excision.

Detailed imaging-acquisition, reading, and nodal-analysis characteristics are summarized in **Table 2**. Five of the six MRI-containing studies used exclusively 3.0-T scanners, whereas Yang et al. used mixed 1.5-T and 3.0-T systems across centers. The two CT studies used thin-section reconstructions (1.00-1.25 mm) and portal venous-phase images. Five of seven studies reported Node-RADS-specific inter-observer agreement, with kappa values ranging from 0.617 to 0.82, while two studies did not report a Node-RADS agreement metric. Reader experience, blinding, adjudication, and target-node selection varied. Most studies used the highest-scoring mesorectal or superior rectal node to represent patient-level status. Only Jiang et al. and Zhuang et al. explicitly included broader regional compartments, including lateral pelvic nodes.

**Table 2:** Technical and methodological characteristics of imaging acquisition and Node-RADS assessment in the included studies.

Study	Imaging modality	Reader number and experience	Reading method	Inter-reader agreement	MRI field strength	MRI slice thickness/key protocol	CT slice thickness	CT contrast phase	Nodal compartment and analysis level
Huang et al. (16)	MRI	Two radiologists; 10 and >15 years of radiology experience (as reported)	Readers evaluated MRI separately and were blinded to pathology; adjudication not reported	Not reported	3.0 T Discovery 750W (GE Healthcare)	High-resolution T2WI: 3 mm, 0-mm gap; axial T1WI, multiplanar high-resolution T2WI, DWI, and contrast-enhanced T1WI; enhanced T1WI acquired 2–3 min after injection	Not applicable	Not applicable	Mesorectal and superior rectal artery nodes; highest Node-RADS score represented each patient (patient-level)
Jiang et al. (9)	CE-CT, T2WI, and contrast-enhanced T1WI	Two readers, each >4 years of abdominal imaging experience; third reader >20 years	Two readers assessed independently and were blinded to staging, pathology, and other examinations; discrepancies adjudicated by reader 3	Weighted kappa: 0.742 (CE-CT), 0.776 (T2WI), 0.798 (T1CE); SAD ICC: 0.974, 0.979, 0.980	3.0 T MAGNETOM Trio (Siemens)	3 mm for T2WI and T1CE; triplanar non-fat-suppressed T2WI; axial delayed contrast-enhanced T1WI (~5 min)	1.25 mm (GE) or 1.00 mm (Siemens)	Portal venous phase (65–70 s)	Mesorectal, superior rectal, inferior mesenteric, internal iliac, obturator, and inferior rectal nodes; highest-scoring node per modality per patient
Niu et al. (12)	CE-CT	Reader 1: 2 years; reader 2: >10 years; reader 3: >20 years of abdominal imaging experience	Two trained readers were blinded to postoperative pathology; disagreements resolved by reader 3	Cohen’s kappa: 0.78 (size-prioritized), 0.71 (morphology-prioritized), 0.82 (Node-RADSmax)	Not applicable	Not applicable	1 mm slice thickness and 1 mm increment	Portal venous phase after bolus tracking	Mesorectal and superior rectal artery nodes; size-prioritized and morphology-prioritized nodes; Node-RADSmax at patient level
Niu et al. (8)	MRI	Two radiologists with >20 and 10 years of pelvic imaging experience	Readers evaluated MRI separately and were blinded to pathology; target node selected through consultation	Cohen’s kappa for Node-RADS: 0.75	3.0 T Discovery 750W (GE Healthcare)	T1WI, high-resolution T2WI, DWI, and venous-phase contrast-enhanced T1WI; detailed thickness in **Supplementary Material**	Not applicable	Not applicable	Mesorectal and superior rectal artery nodes; highest Node-RADS score represented each patient
Xue et al. (13)	MRI	Node-RADS readers: 2 and 15 years of rectal imaging experience; standardized training by a senior radiologist	Independent blinded assessment; disagreements resolved with a senior radiologist; repeat scoring after 6 months	Inter-observer weighted kappa 0.617 (95% CI 0.541-0.693); intra-observer weighted kappa 0.788 (0.722-0.854)	3.0 T Skyra (Siemens)	Sagittal, coronal, and axial T2WI; T1WI, DWI, and high-resolution oblique axial T2WI; thickness in **Supplementary Material**	Not applicable	Not applicable	Mesorectal nodes; highest Node-RADS score per patient
Yang et al. (15)	MRI	Reader 1: 4 years; reader 2: >10 years; reader 3: >20 years of rectal MRI experience	Readers initially assessed independently, then conferred to identify target node and score; discrepancies resolved by reader 3	Node-RADS agreement not reported; reported ICC concerned radiomics ROI reproducibility	Mixed 1.5 T and 3.0 T scanners across three centers	CET1, T2WI, and DWI; acquisition parameters in **Supplementary Material**; 1 x 1 x 1 mm3 was radiomics resampling, not slice thickness	Not applicable	Not applicable	Mesorectal nodes; size- and shape-priority nodes, maximum score per patient; pre-nCRT MRI with post-nCRT pathology
Zhuang et al. (14)	MRI	Readers 1 and 2: 4 and 6 years; reader 3: 20 years of abdominal oncologic imaging experience	Independent mutually blinded assessment; standardized training and 30 test cases; adjudication by reader 3	Weighted Cohen’s kappa for 5-category Node-RADS: 0.765 (95% CI 0.696-0.834); margin 0.673, texture 0.771, shape 0.814	3.0 T MAGNETOM Trio (Siemens)	Triplanar non-fat-suppressed T2WI, DWI, and T1WI; representative high-resolution T2 slice thickness 3 mm	Not applicable	Not applicable	Mesorectal, superior rectal, inferior mesenteric, internal iliac, obturator, and inferior rectal nodes; highest-scoring node per patient

CE-CT, contrast-enhanced computed tomography; CET1/T1CE, contrast-enhanced T1-weighted imaging; DWI, diffusion-weighted imaging; ICC, intraclass correlation coefficient; MRI, magnetic resonance imaging; nCRT, neoadjuvant.

### 3.3 Threshold effect and heterogeneity test

**Figure 3:**
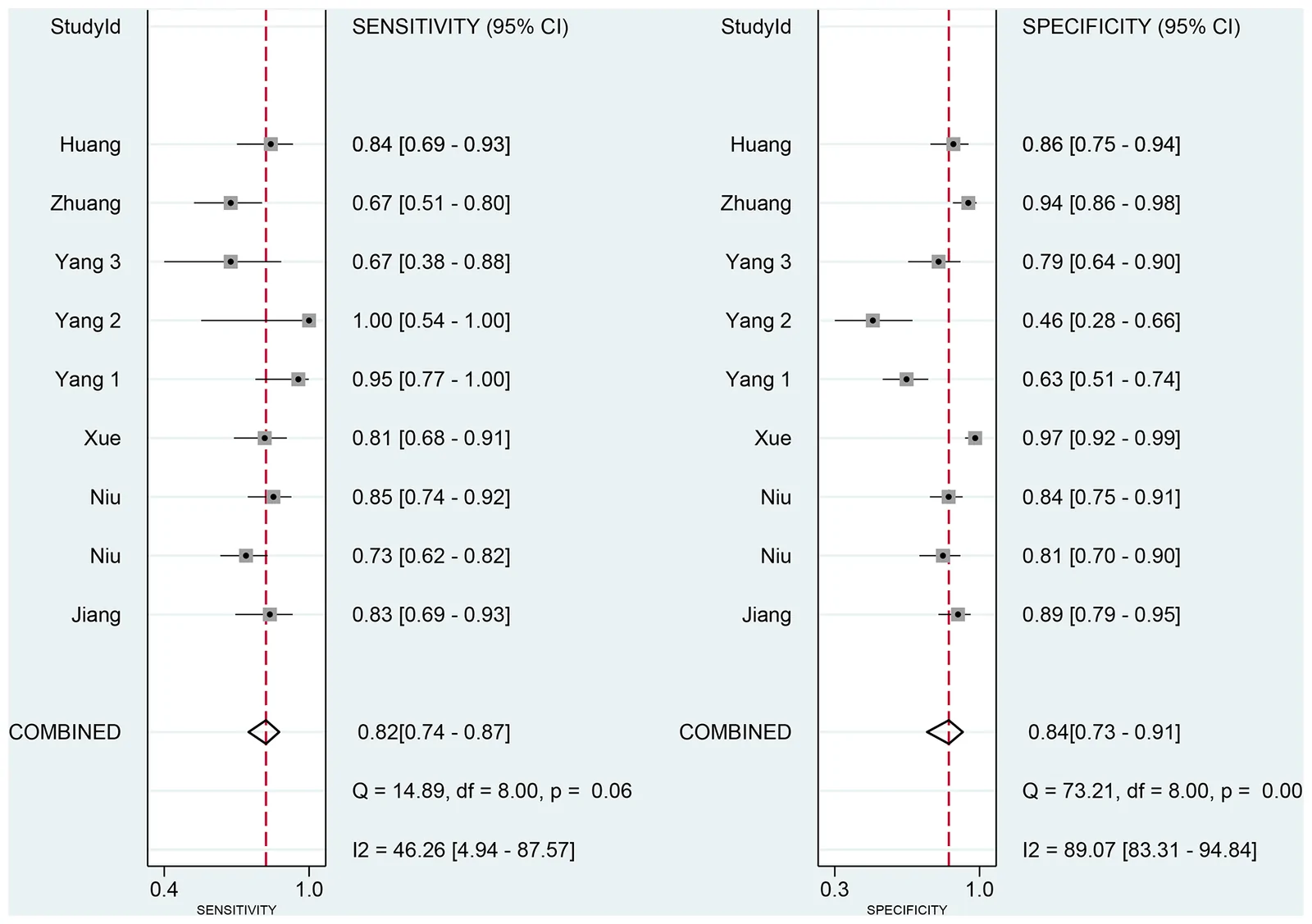
Forest plots of pooled sensitivity and specificity of Node-RADS for detecting lymph node metastasis in rectal cancer.

**Figure 4:**
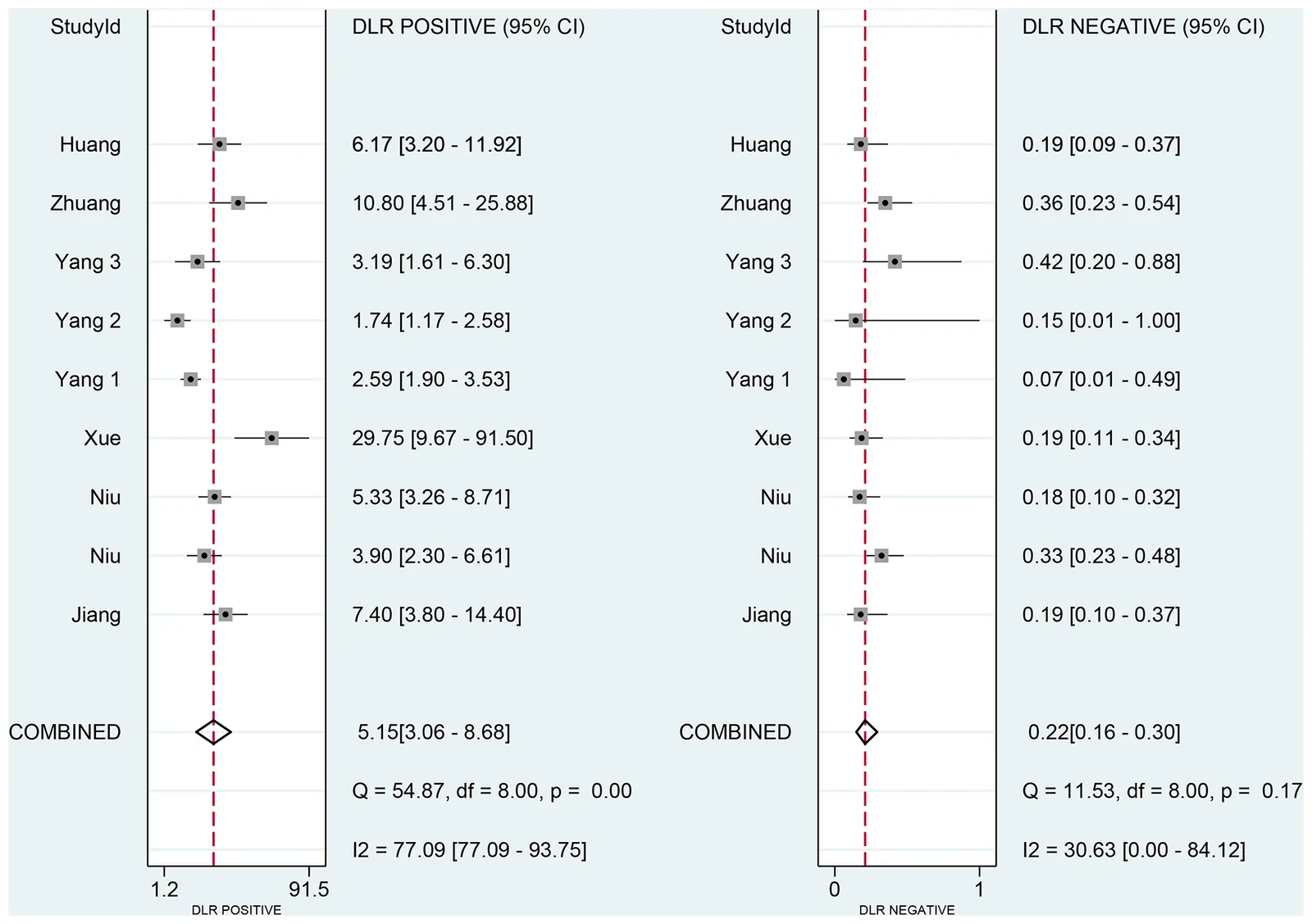
Forest plots of pooled positive likelihood ratio (PLR) and negative likelihood ratio (NLR) of Node-RADS for detecting lymph node metastasis in rectal cancer.

**Figure 5:**
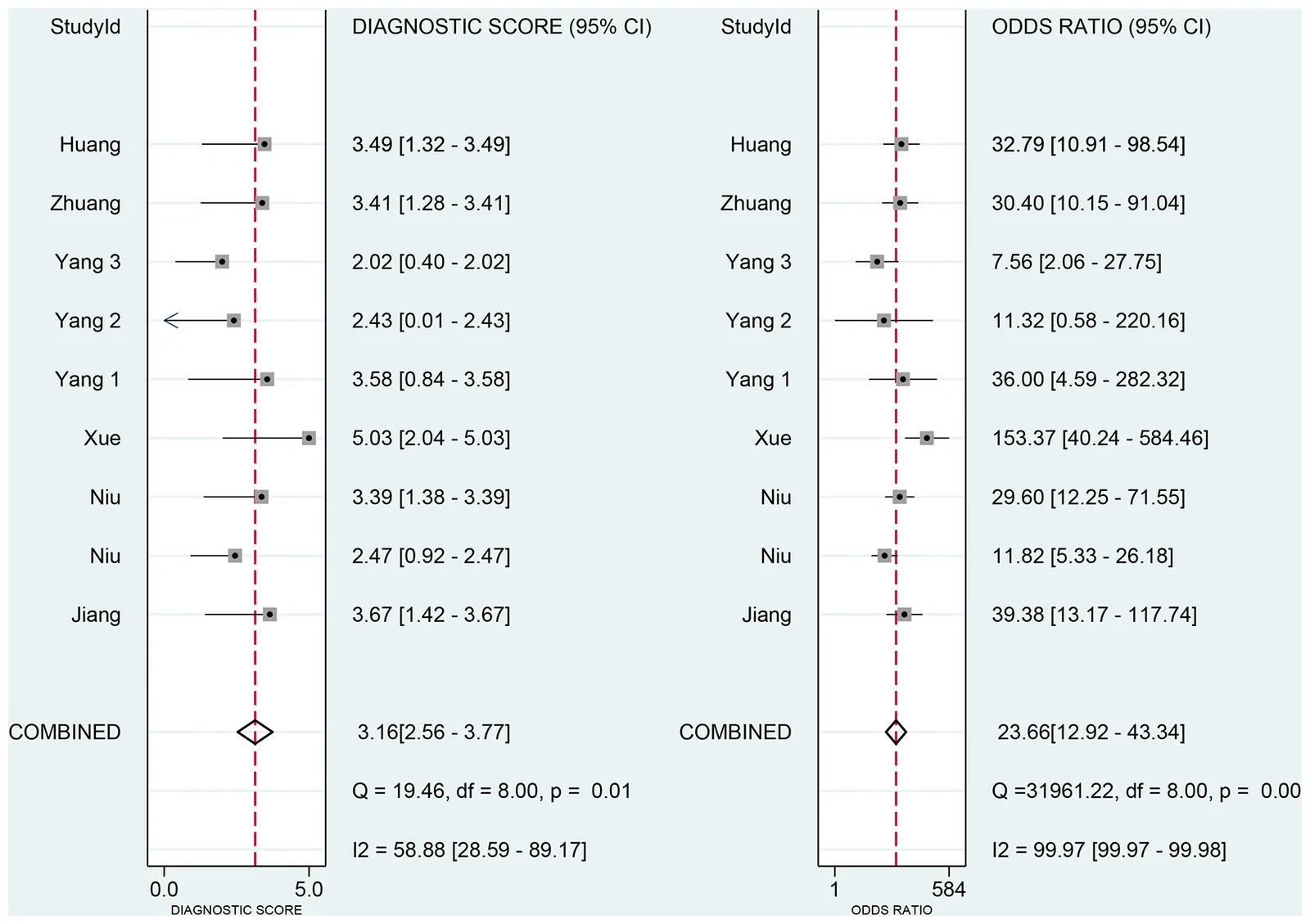
Forest plots of diagnostic score and diagnostic odds ratio (DOR) of Node-RADS for detecting lymph node metastasis in rectal cancer.

The threshold-effect test yielded a Spearman correlation coefficient of 0.477 (P = 0.194), indicating no statistically detectable threshold effect. Nevertheless, clinically relevant dichotomization varied across studies, particularly regarding whether Node-RADS category 3 was considered positive. Heterogeneity was moderate for sensitivity (I²=46.26%, P = 0.06) and substantial for specificity (I²=89.07%, P<0.001), as shown in **Figure 3**. PLR and DOR also demonstrated significant heterogeneity (I²=77.09% and 99.97%, respectively; **Figures 4**, **5**). Given the clinical and methodological variability in treatment setting, imaging acquisition, reader workflow, nodal compartment, analysis level, and threshold selection, pooled estimates were generated with a bivariate random-effects model.

### 3.4 Pooled diagnostic performance

**Figure 6:**
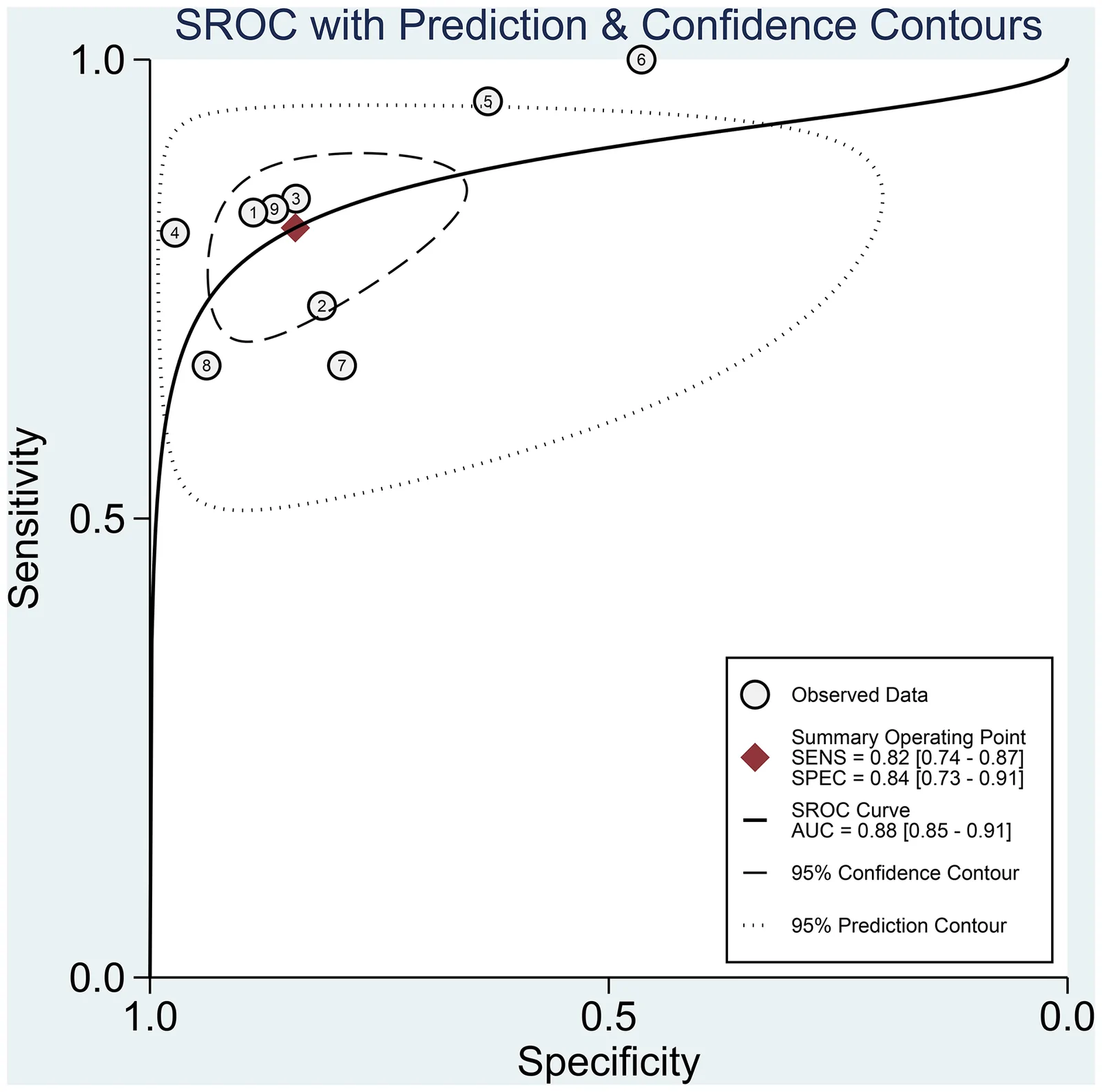
Summary receiver operating characteristic (SROC) curve of Node-RADS for detecting lymph node metastasis in rectal cancer.

A bivariate random-effects model was leveraged to aggregate the diagnostic performance of Node-RADS for lymph node metastasis of RC. As shown in **Figures 3**–**5**, the overall sensitivity, specificity, PLR, NLR, and DOR were 0.82 (95% CI: 0.74–0.87), 0.84 (0.73–0.91), 5.15 (3.06–8.68), 0.22 (0.16–0.30), and 23.66 (12.92–43.34), respectively. As shown in **Figure 6**, the SROC curve analysis resulted in an AUC of 0.88 (0.85–0.91). These findings indicated that Node-RADS had good diagnostic accuracy and clinical discriminatory ability for lymph node metastasis in RC.

**Figure 7:**
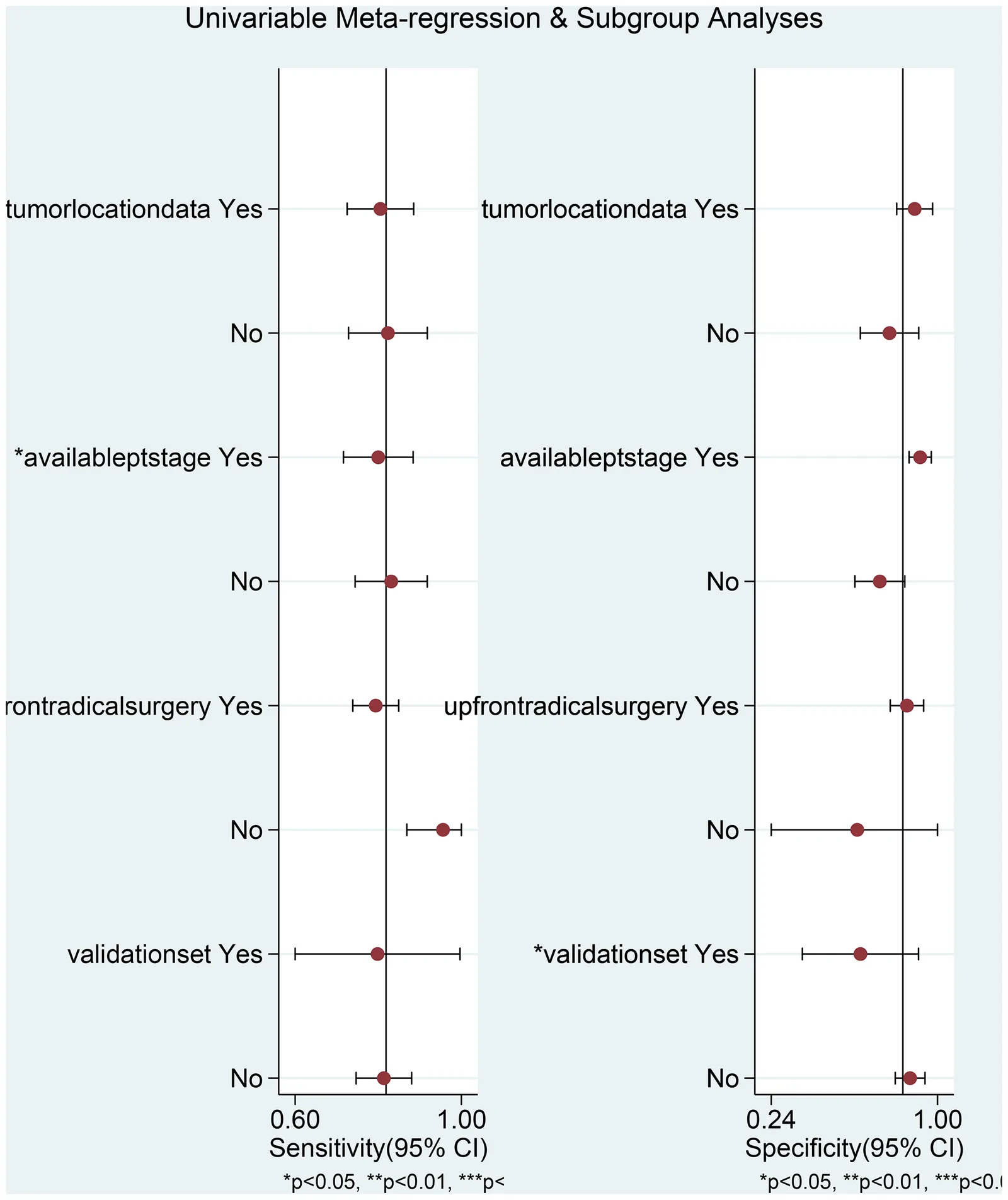
Meta-regression analysis exploring potential sources of heterogeneity.

In the CT subgroup, pooled sensitivity, specificity, PLR, NLR, and DOR were 0.76 (95% CI: 0.71-0.81), 0.80 (0.67-0.88), 3.7 (2.2-6.4), 0.30 (0.24-0.38), and 12 (6-25), respectively. Corresponding estimates for the MRI subgroup were 0.82 (0.75-0.88), 0.86 (0.76-0.93), 6.0 (3.4-10.5), 0.20 (0.15-0.28), and 30 (17-52). Although the MRI estimates were numerically higher for several measures, no formal interaction test was performed because the subgroups contained few studies and Jiang et al. contributed correlated multimodal estimates from the same participants. Therefore, these results should be viewed as descriptive and do not establish MRI superiority over CT. Jiang et al. additionally reported that Node-RADSmax favored sensitivity whereas Node-RADSmin favored specificity, suggesting complementary rather than necessarily superior modality information. Meta-regression did not identify tumor-location reporting, pathological T-stage reporting, direct radical surgery, or validation-set use as clear and stable sources of heterogeneity (**Figure 7**).

### 3.5 Publication bias and sensitivity analyses

**Figure 8:**
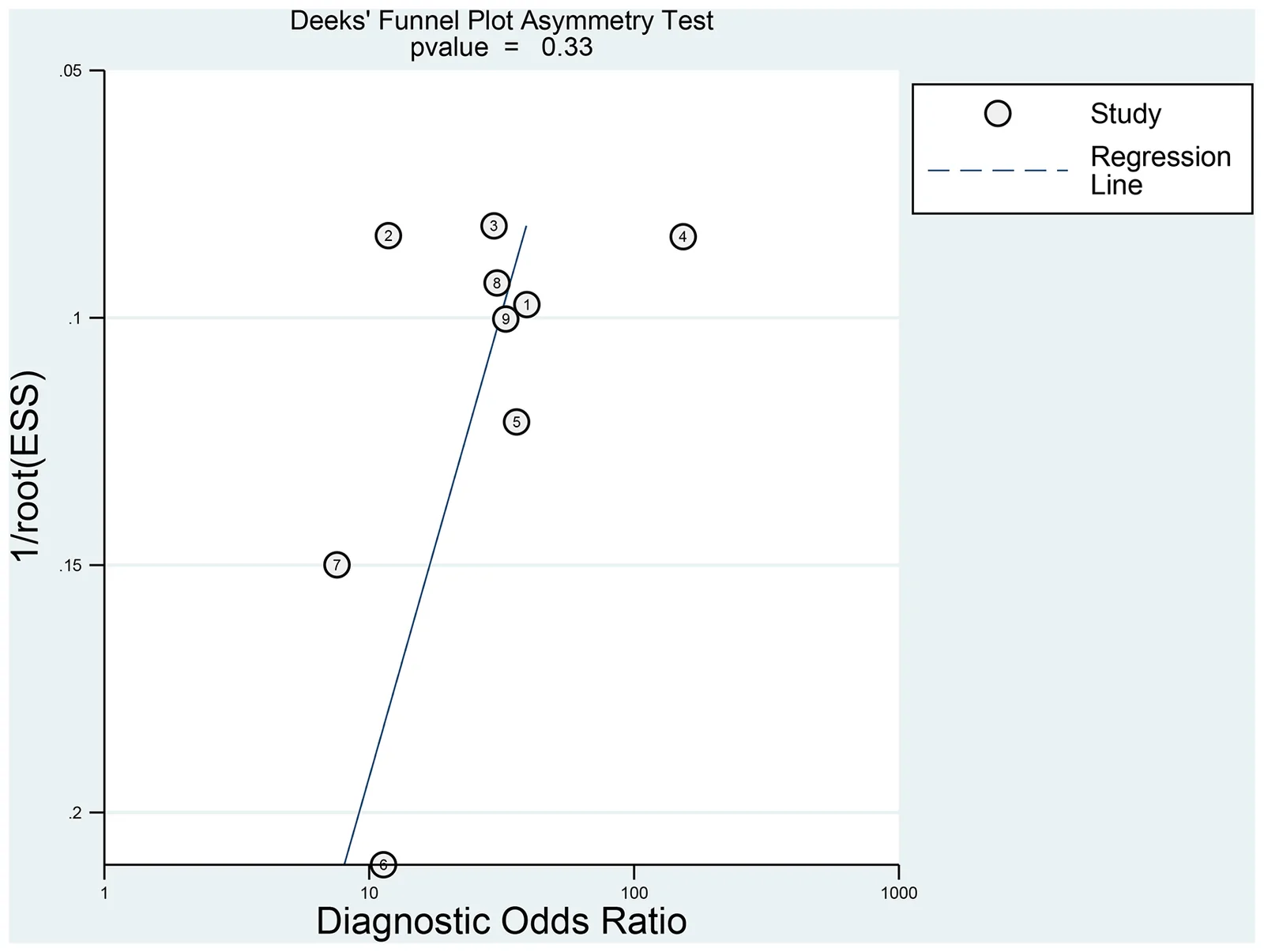
Deeks’ funnel plot for assessment of publication bias.

**Figure 9:**
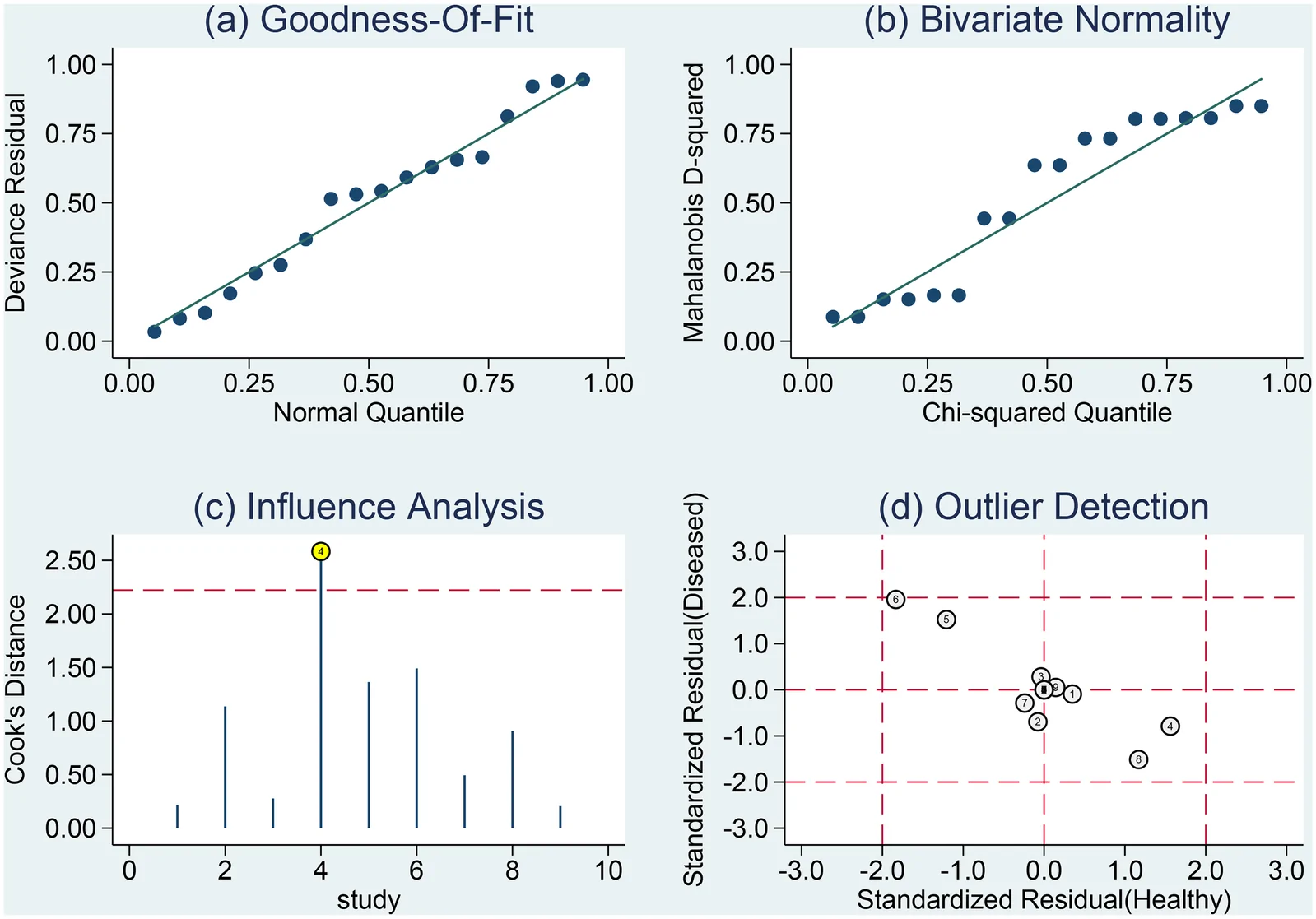
Model diagnostic plots: **(a)** goodness-of-fit; **(b)** bivariate normality; **(c)** influence analysis; **(d)** outlier detection.

Deeks’ funnel plot was approximately symmetrical and the quantitative test yielded P = 0.33 (**Figure 8**). This finding did not identify statistically significant small-study effects, but it could not exclude publication bias because only seven studies were available. Goodness-of-fit and bivariate normality analyses (**Figures 9A, B**) supported model adequacy. Influence analysis identified one potentially influential study, whereas formal outlier detection identified none (**Figures 9C, D**). Excluding the influential study produced pooled sensitivity, specificity, PLR, NLR, and DOR of 0.83 (95% CI: 0.73-0.90), 0.81 (0.71-0.88), 4.4 (2.9-6.5), 0.21 (0.14-0.32), and 21 (13-34), respectively, without a material change in the overall conclusion.

**Figure 10:**
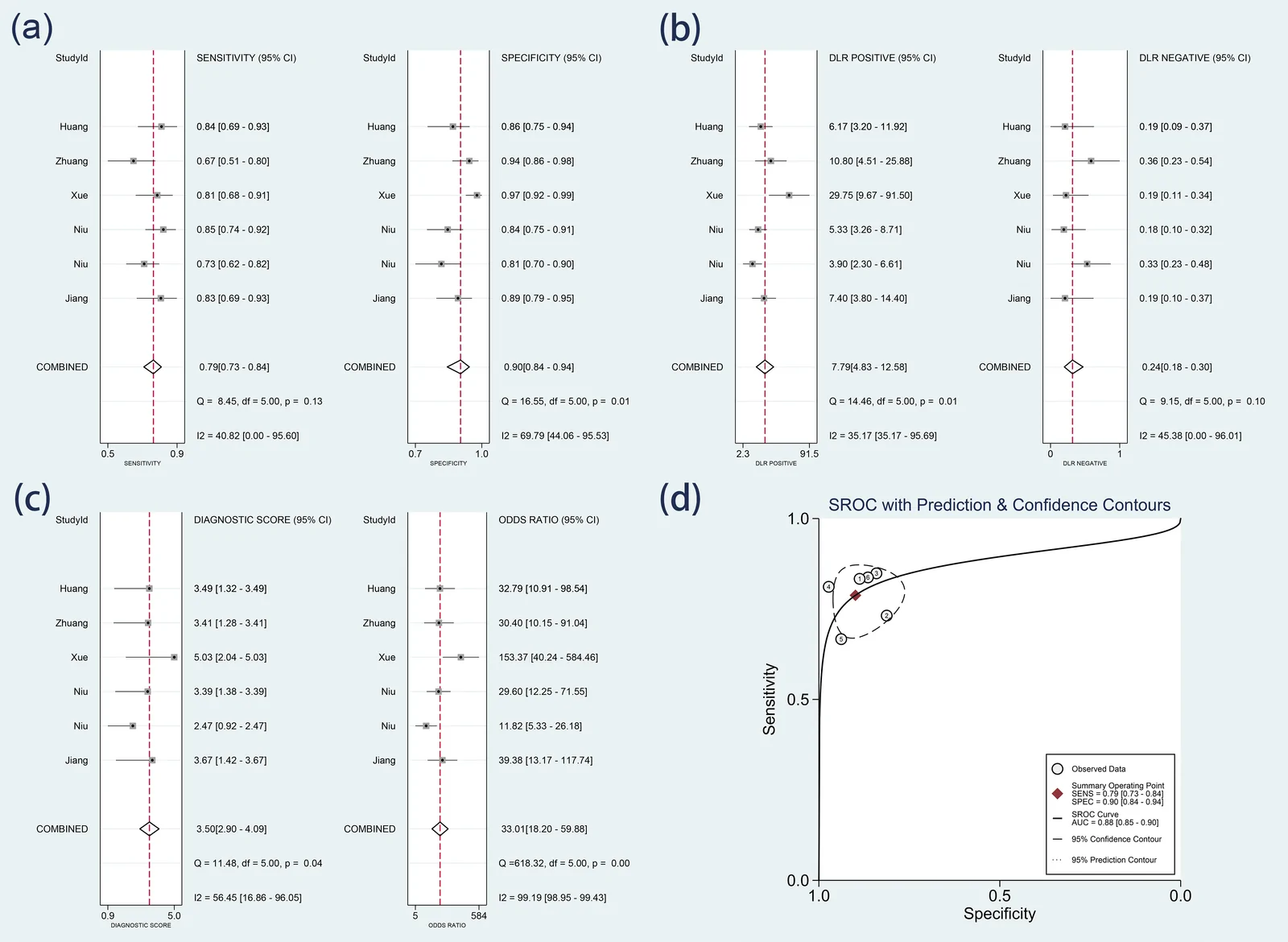
**(A)** Forest plots of pooled sensitivity and specificity in the sensitivity analysis excluding Yang et al. (2026). **(B)** Forest plots of pooled positive likelihood ratio (PLR) and negative likelihood ratio (NLR) in the sensitivity analysis excluding Yang et al. (2026). **(C)** Forest plots of diagnostic score and diagnostic odds ratio (DOR) in the sensitivity analysis excluding Yang et al. (2026). **(D)** Summary receiver operating characteristic (SROC) curve with confidence and prediction contours in the sensitivity analysis excluding Yang et al. (2026).

A clinically focused sensitivity analysis then excluded Yang et al. (2026), leaving six treatment-naive studies with 804 patients (331 with and 473 without lymph node metastasis). The pooled sensitivity, specificity, PLR, NLR, and DOR were 0.79 (95% CI: 0.73-0.84), 0.90 (0.84-0.94), 7.79 (4.83-12.58), 0.24 (0.18-0.30), and 33.01 (18.20-59.88), respectively, and the area under the SROC curve (AUC) remained 0.88 (0.85-0.90). Sensitivity heterogeneity decreased from I²=46.26% to 40.82%, and specificity heterogeneity decreased from I²=89.07% to 69.79%, although residual heterogeneity remained substantial. Comparisons with the primary analysis are shown in **Table 3**, and the corresponding forest plots and SROC curve are presented in **Figures 10A–D**. These results indicated stable overall discrimination while suggesting that the post-treatment Yang cohort contributed to specificity-related heterogeneity.

**Table 3:** Comparison of primary pooled results with the sensitivity analysis excluding Yang et al. (15).

Diagnostic measure	Primary analysis	Sensitivity analysis excluding Yang et al. (15)	Interpretation
Sensitivity (95% CI)	0.82 (0.74–0.87)	0.79 (0.73–0.84)	Slightly decreased; 95% CIs overlapped, indicating broadly stable sensitivity.
Specificity (95% CI)	0.84 (0.73–0.91)	0.90 (0.84–0.94)	Increased, with improved specificity after exclusion.
PLR (95% CI)	5.15 (3.06–8.68)	7.79 (4.83–12.58)	Increased, indicating stronger rule-in performance.
NLR (95% CI)	0.22 (0.16–0.30)	0.24 (0.18–0.30)	Slightly increased; rule-out performance was broadly unchanged.
DOR (95% CI)	23.66 (12.92–43.34)	33.01 (18.20–59.88)	Increased, indicating improved overall diagnostic discrimination.
SROC AUC (95% CI)	0.88 (0.85–0.91)	0.88 (0.85–0.90)	Essentially unchanged, indicating stable overall discrimination.
Sensitivity heterogeneity, I²	46.26%	40.82%	Modestly decreased, indicating slightly lower heterogeneity.
Specificity heterogeneity, I²	89.07%	69.79%	Markedly decreased but remained substantial.

AUC, area under the curve; CI, confidence interval; DOR, diagnostic odds ratio; NLR, negative likelihood ratio; PLR, positive likelihood ratio; SROC, summary receiver operating characteristic.

### 3.6 Clinical utility

**Figure 11:**
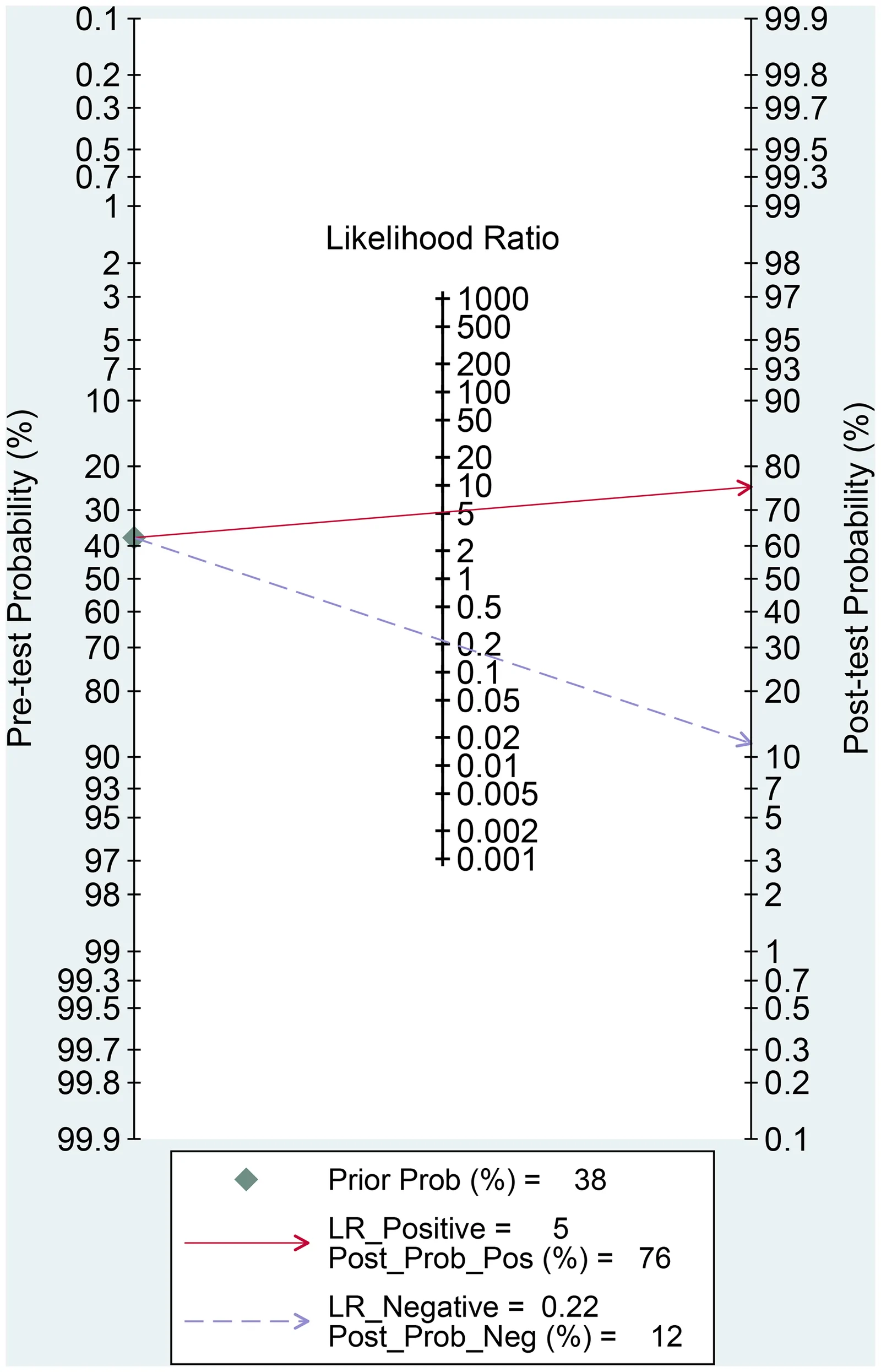
Fagan nomogram based on the observed prevalence of lymph node metastasis of 37.6% among included participants (rounded to 38% for plotting). A positive Node-RADS result increased the post-test probability to approximately 76%, whereas a negative result reduced it to approximately 12%.

The Fagan plot was used to evaluate clinical utility (**Figure 11**). The pre-test probability was set to the observed prevalence of lymph node metastasis among all included participants (374/994, 37.6%; rounded to 38% in the nomogram). Using the pooled likelihood ratios (PLR 5.15, displayed as 5.0 in the nomogram; NLR 0.22), a positive Node-RADS result increased the post-test probability to approximately 76%, whereas a negative result reduced it to approximately 12%. Thus, Node-RADS meaningfully changed disease probability, but the remaining post-test uncertainty indicated that results should be integrated with tumor stage, other high-risk MRI features, and multidisciplinary assessment rather than used alone.

## 4 Discussion

This meta-analysis systematically evaluated Node-RADS for detecting lymph node metastasis in RC. In the primary analysis, pooled sensitivity, specificity, PLR, NLR, and AUC were 0.82, 0.84, 5.15, 0.22, and 0.88, respectively. Exclusion of the clinically distinct post-neoadjuvant cohort left the AUC unchanged at 0.88 and increased pooled specificity to 0.90, while reducing specificity heterogeneity. At the observed disease prevalence of 37.6%, a positive result increased the post-test probability to approximately 76% and a negative result reduced it to approximately 12%. Collectively, these findings support Node-RADS as a useful standardized adjunct, but not as an isolated determinant of treatment.

The findings are broadly consistent with the pan-cancer meta-analysis by Lu et al. (10), which includes 13 studies and 1,341 participants across multiple tumor types and reports pooled sensitivity, specificity, and hierarchical SROC (HSROC) AUC of 0.79, 0.86, and 0.90. Lu et al. used an HSROC framework, whereas the present study used the closely related bivariate random-effects model to jointly pool sensitivity and specificity and derive an SROC curve. The more important methodological difference is the population. Lu et al. combined tumors with different lymphatic drainage pathways, nodal size distributions, imaging protocols, and pathological behavior, while our eligibility criteria were restricted to RC and postoperative pathological verification. This narrower scope improves disease-specific interpretability but includes only seven studies and therefore reduces statistical power for meta-regression, publication-bias assessment, and formal subgroup interaction testing. Moreover, the CT-MRI comparisons in both meta-analyses are indirect and potentially confounded. In our data, the within-patient multimodal estimates from Jiang et al. are correlated and cannot be treated as independent evidence. Accordingly, numerical modality differences should not be interpreted as proof of superiority.

The pooled estimates also align with individual RC studies. MRI-based Node-RADS outperformed ESGAR and short-axis criteria in Niu et al. (8), and CT-based Node-RADS outperformed short diameter in Niu et al. (12). Jiang et al. reported similar AUCs across contrast-enhanced CT, T2WI, and contrast-enhanced T1WI in the same patients (9). The descriptive technical summary in **Table 2**, however, shows variation in reader experience, target-node selection, adjudication, MRI field strength, and nodal compartment. CT protocols were relatively consistent (thin-section portal venous-phase imaging), whereas MRI sequence details and slice thickness were incompletely reported in several studies. These factors may affect visibility of small nodes, assessment of borders and internal texture, and inter-reader reproducibility. Because each technical covariate was available in too few studies, formal meta-regression would have been underpowered and prone to overfitting. The residual heterogeneity should therefore be regarded as unexplained rather than attributed to a single modality.

Some individual studies reported higher performance than the pooled estimate. Xue et al. reported an MRI-based Node-RADS AUC of 0.912 and an association with disease-free survival (13), suggesting possible prognostic value. Yang et al. evaluated Node-RADS in a fundamentally different context of locally advanced RC after neoadjuvant chemoradiotherapy, when nodal shrinkage, fibrosis, signal alteration, and residual micrometastases may disrupt conventional morphology-based assessment (15, 17). Retaining that study in the primary analysis broadened the clinical scope but increased heterogeneity and limited direct generalization to treatment-naive staging. The newly added sensitivity analysis excluding Yang et al. showed an unchanged AUC, higher specificity, PLR, and DOR, and lower specificity heterogeneity (**Table 3**; **Figures 10A–D**). This supports robustness of overall discrimination while confirming that initial staging and post-treatment restaging should be interpreted as distinct clinical applications.

Beyond differences in study populations and treatment settings, the favorable diagnostic performance of Node-RADS may partly be attributed to its integration of lymph node size and morphological features into a structured risk-stratification system. Conventional imaging assessment is often based on the short-diameter criterion. However, metastatic lymph nodes in RC are not necessarily significantly enlarged, while inflammatory or reactive hyperplastic lymph nodes may also present with increased size. Thus, relying solely on size can easily lead to missed or incorrect diagnoses. Node-RADS employs a stepwise approach to assess lymph nodes. First, lymph nodes are classified as normal, enlarged, or mass-like lymph nodes based on size thresholds for distinct anatomical regions. Then, morphological features like internal structure, margins, and shape are further evaluated. Finally, the integrated information of size and morphology yields a risk category from 1 to 5 points. For this risk category, 1 point indicates a very low risk of metastasis and 5 points indicate a very high risk (6). In the appraisement of lymph nodes of RC, features like increased short diameter, internal heterogeneity or necrosis, irregular or indistinct margins, spherical shape, and loss of the fatty hilum may reflect tumor cell infiltration, extranodal extension, or disruption of the internal nodal architecture. Therefore, rather than relying solely on lymph node size, Node-RADS combines “size” and “malignant morphological features” to assess metastatic risk, thereby providing a more comprehensive reflection of the likelihood of nodal malignancy compared with conventional short-diameter criteria (6, 8).

Another advantage of Node-RADS is its potential to standardize risk communication. Conventional imaging reports often use subjective terms such as “suspicious,” “slightly enlarged,” or “ill-defined,” which may lead to inconsistent interpretation between radiologists and clinical teams. By assigning lymph nodes to five ordered risk categories, Node-RADS provides a clearer and more reproducible description of the likelihood of nodal involvement (6, 8). However, the score should not be used alone to determine treatment. In clinical practice, Node-RADS findings should be interpreted together with established tumor- and patient-related factors, including T stage, extramural venous invasion (EMVI), mesorectal fascia (MRF) involvement, tumor location, serum tumor markers, and the anatomical compartment of the suspicious node, within a multidisciplinary framework (3, 18). Thus, high or low scores may appropriately increase or decrease clinical suspicion, but decisions regarding neoadjuvant therapy, surgery, or follow-up should remain based on comprehensive assessment rather than a single imaging score.

A clinically important issue concerns the interpretation of Node-RADS category 3. Category 3 represents intermediate risk, and the included studies did not use a uniform binary definition: some treated scores ≥3 as positive, whereas others treated scores ≥4 as positive or selected an optimal cutoff *post hoc*. Classifying category 3 as positive generally favors sensitivity at the expense of specificity; classifying it as negative has the opposite effect. Although no statistical threshold effect was detected, the small number of studies limits the power of this test, and threshold variability may still contribute to heterogeneity and optimistic within-study performance. Clinically, category 3 should not automatically trigger or exclude neoadjuvant treatment. It should be interpreted in conjunction with the stage and histological grade of the primary tumor, as recommended in the Node-RADS framework, as well as other clinically relevant risk features within a multidisciplinary setting (6, 7). Accordingly, the clinical interpretation of category 3 remains uncertain and requires external validation.

A separate issue concerns the anatomical generalizability of the pooled estimates. Most included studies focused on mesorectal and superior rectal lymph nodes, whereas only Jiang et al. and Zhuang et al. explicitly included lateral pelvic compartments such as the internal iliac and obturator nodes (9, 14). None reported separate diagnostic 2 × 2 data for mesorectal versus lateral pelvic nodes, precluding compartment-specific pooling. This distinction is clinically relevant because ESGAR recommendations apply specific size and morphological criteria to lateral pelvic nodes (3), while the JSCCR guidelines consider lateral nodal involvement in lower rectal cancer a separate management issue with potential implications for lateral lymph node dissection (18). Therefore, the pooled estimates should not be assumed to apply equally to mesorectal and lateral pelvic nodes. Compartment-specific validation is required before these pooled estimates can be generalized to both nodal regions.

Beyond these clinical interpretive and anatomical considerations, several methodological limitations require emphasis. First, heterogeneity was substantial for specificity, PLR, and DOR. **Table 2** identifies plausible contributors, including reader experience, blinding and adjudication, MRI field strength, slice thickness, target-node selection, nodal compartment, and patient-level versus node-level analysis. However, systematic meta-regression of these factors was not statistically defensible because only seven studies were available, several variables were missing, MRI field strength and CT contrast phase showed little variation, and study-level covariates would have consumed excessive degrees of freedom. The decrease in specificity heterogeneity after excluding Yang et al. suggests that treatment setting contributed, but the remaining I² of 69.79% indicates that technical and methodological variability persists. Second, the index-test domain of QUADAS-2 was frequently rated high risk because most studies chose the positivity threshold after inspecting their own ROC data, commonly using the Youden index. Such data-driven optimization maximizes apparent sensitivity and specificity in the development sample and can produce optimism bias, especially without independent validation. Consequently, the pooled accuracy may overestimate real-world performance and may not transport directly across centers or patient populations. Variation between thresholds of ≥3 and ≥4 also changes the sensitivity-specificity trade-off and complicates a single pooled binary interpretation. The results should therefore be considered supportive evidence for structured reporting rather than validation of one universal treatment threshold. Third, most studies used patient-level pN status rather than rigorous node-by-node imaging-pathology matching. The highest-scoring imaged node may not be the node that was pathologically metastatic, creating attribution error and potentially inflating or attenuating diagnostic estimates. Inter-observer agreement was also incompletely and inconsistently reported. Five studies provided Node-RADS kappa values (0.617-0.82), whereas two did not. Reader experience and adjudication procedures varied, and different kappa formulations prevented meaningful pooling. All included studies were retrospective and predominantly from Chinese centers, which limits geographic and prospective generalizability. Finally, with only seven studies, Deeks’ funnel plot has low power. P = 0.33 should be interpreted as no detected asymmetry, not proof that publication bias is absent.

Future large-scale, multicenter prospective studies should use standardized CT/MRI acquisition protocols, predefined Node-RADS thresholds, independent external validation cohorts, and explicit reporting of reader training and inter-observer agreement. Whenever feasible, imaging findings should be matched to pathology on a node-by-node or clearly compartment-specific basis. Studies should separately evaluate initial staging and post-treatment restaging, report compartment-specific diagnostic performance for mesorectal and lateral pelvic nodes, and prespecify how category 3 is classified and incorporated into clinical management. Integration of Node-RADS with clinical indicators, serum tumor markers, EMVI, MRF status, radiomics, deep learning, and explainable artificial intelligence may further improve accuracy and interpretability (15, 19), but such models require prospective calibration and clinical-impact evaluation before implementation.

## 5 Conclusion

In conclusion, Node-RADS demonstrates good pooled diagnostic discrimination for lymph node metastasis in RC, and sensitivity analysis restricted to treatment-naive cohorts supports the stability of the overall AUC. It may serve as a standardized imaging-based adjunct to preoperative nodal assessment and has the potential to improve the consistency of clinical risk stratification. Nevertheless, substantial residual heterogeneity, *post hoc* threshold selection, incomplete inter-reader reporting, patient-level pathological reference standards, and limited evidence for lateral pelvic nodes indicate that further validation is needed before Node-RADS can be used independently for treatment decision-making. Large multicenter prospective studies with prespecified thresholds are warranted.

## Data Availability

The original contributions presented in the study are included in the article/**Supplementary Material**. Further inquiries can be directed to the corresponding author.
