# Supplementary material for: Diagnostic accuracy of Node-RADS for lymph node metastasis in rectal cancer: a systematic review and meta-analysis
Source: Front Oncol. 2026 Aug 26;16:1899172. doi: 10.3389/fonc.2026.1899172 (PMC13553743; doi:10.3389/fonc.2026.1899172)
Supplement: Supplementary file 1 [file DataSheet1.pdf]

Pubmed	
#	Query
1	Rectal Neoplasms[MeSH Terms]
2	"Cancer of Rectum"[Title/Abstract] OR "Cancer of the Rectum"[Title/Abstract] OR "neoplasm of the rectum"[Title/Abstract] OR "neoplasma recti"[Title/Abstract] OR "pararectal tumo*r"[Title/Abstract] OR "Rectal Cancer*"[Title/Abstract] OR "rectal mass tumor"[Title/Abstract] OR "rectal neoplas*"[Title/Abstract] OR "rectal tumo*r*"[Title/Abstract] OR "Rectum Cancer*"[Title/Abstract] OR "rectum mass tumor"[Title/Abstract] OR "rectum neoplas*"[Title/Abstract] OR "rectum tumo*r"[Title/Abstract] OR "retrorectal tumo*r"[Title/Abstract] OR "tumo*r of the rectum"[Title/Abstract] OR "tumo*r recti"[Title/Abstract]
3	#1 OR #2
4	"Node-RADS"[Title/Abstract] OR "Node Reporting and Data System"[Title/Abstract]
5	#3 AND #4

Embase	
#	Query
1	'rectum tumor'/exp
2	'cancer of rectum':ti,ab,kw OR 'cancer of the rectum':ti,ab,kw OR 'neoplasm of the rectum':ti,ab,kw OR 'neoplasma recti':ti,ab,kw OR 'pararectal tumo*r':ti,ab,kw OR 'rectal cancer*':ti,ab,kw OR 'rectal mass tumor':ti,ab,kw OR 'rectal neoplas*':ti,ab,kw OR 'rectal tumo*r*':ti,ab,kw OR 'rectum cancer*':ti,ab,kw OR 'rectum mass tumor':ti,ab,kw OR 'rectum neoplas*':ti,ab,kw OR 'rectum tumo*r':ti,ab,kw OR 'retrorectal tumo*r':ti,ab,kw OR 'tumo*r of the rectum':ti,ab,kw OR 'tumo*r recti':ti,ab,kw
3	#1 OR #2
4	'node-rads':ti,ab,kw OR 'node reporting and data system':ti,ab,kw
5	#3 AND #4

Cochrane Library	
#	Query
1	MeSH descriptor: [Rectal Neoplasms] explode all trees
2	(‘Cancer of Rectum’ OR ‘Cancer of the Rectum’ OR ‘neoplasm of the rectum’ OR ‘neoplasma recti’ OR ‘pararectal tumo*r’ OR ‘Rectal Cancer*’ OR ‘rectal mass tumor’ OR ‘rectal neoplas*’ OR ‘rectal tumo*r*’ OR ‘Rectum Cancer*’ OR ‘rectum mass tumor’ OR ‘rectum neoplas*’ OR ‘rectum tumo*r’ OR ‘retrorectal tumo*r’ OR ‘tumo*r of the rectum’ OR ‘tumo*r recti’):ti,ab,kw
3	#1 OR #2
4	('Node-RADS' OR 'Node Reporting and Data System'):ti,ab,kw
5	#3 AND #4

Web of Science	
#	Query
1	TS=((Cancer of Rectum) OR (Cancer of the Rectum) OR (neoplasm of the rectum) OR (neoplasma recti) OR (pararectal tumo*r) OR (Rectal Cancer*) OR (rectal mass tumor) OR (rectal neoplas*) OR (rectal tumo*r*) OR (Rectum Cancer*) OR (rectum mass tumor) OR (rectum neoplas*) OR (rectum tumo*r) OR (retrorectal tumo*r) OR (tumo*r of the rectum) OR (tumo*r recti))
2	TS=((Node Reporting and Data System) OR (Node-RADS))
3	#2 AND #1

VIP	
#	Query
1	(任意字段=直肠癌 OR 任意字段=直肠肿瘤) AND 任意字段=淋巴结报告和数据系

	统)

CNKI	
#	Query
1	（篇关摘：直肠癌 + 直肠肿瘤(精确)）AND（篇关摘：淋巴结报告和数据系统+ node-rads"(精确)）

Wanfang	
#	Query
1	全部:(直肠癌 or 直肠肿瘤) and 全部:(node-rads or 淋巴结报告和数据系统)

Chinese Biomedical Literature Database (CBM)	
#	Query
1	("淋巴结报告和数据系统"[常用字段:智能] OR "node-rads"[常用字段:智能]) AND ("直肠癌"[常用字段:智能] OR "直肠肿瘤"[常用字段:智能])
